# Foreword to the special virtual issue dedicated to the proceedings of the PhotonMEADOW2023 Joint Workshop

**DOI:** 10.1107/S1600577524008816

**Published:** 2024-10-11

**Authors:** Elke Plönjes, Jan Grünert, Pavle Juranić, Kai Tiedtke, Marco Zangrando

**Affiliations:** a Deutsches Elektronen-Synchrotron DESY Notkestrasse 85 22607Hamburg Germany; b European XFEL Holzkoppel 4 22869Schenefeld Germany; c SwissFEL Paul Scherrer Institut 5232Villigen Switzerland; d Istituto Officina dei Materiali – CNR, SS 14 – km 163.5, Basovizza, 34149Trieste, Italy; e Elettra Sincrotrone Trieste SCpA SS 14 – km 163.5 Basovizza 34149Trieste Italy

**Keywords:** PhotonMEADOW2023 workshop, free-electron lasers, photon beam diagnostics

## Abstract

Foreword to the virtual issue papers from the PhotonMEADOW2023 workshop.

The virtual focused issue of *Journal of Synchrotron Radiation* (https://journals.iucr.org/special_issues/2024/photonmeadow23) collects a selection of contributions presented at the PhotonMEADOW2023 workshop hosted by Elettra Sincrotrone Trieste at the International Centre for Theoretical Physics on 12–14 September 2023. The event merged two workshops: ‘MEADOW – 10 years after’ and ‘PhotonDiag2023’. The former paid tribute to the 10th anniversary of the original MEADOW2013, the ‘MEtrology, Astronomy, Diagnostics and Optics Workshop’ that was held at the same venue in 2013. The latter represented the 6th edition of the FELS OF EUROPE ‘Workshop on FEL Photon Diagnostics, Instrumentation and Beamline Design’. As they both fell in the same year and brought together an exciting community centred around highest performance photon beamlines for X-ray free-electron lasers (FELs) and synchrotron sources, it was decided to hold both workshops together.

‘MEADOW – 10 years after’, in particular, intended to celebrate the original MEADOW workshop, which back in 2013 represented a unique occasion where the X-ray metrology, the X-ray optics and the X-ray diagnostics communities got together for the first time, gathering in the same place people coming from synchrotrons, free-electron lasers, universities, companies, astronomical observatories, *etc*. That event served also as the starting and driving force to realize new pioneering projects like the X-ray optics database (DABAX) and the now worldwide spread suite for optical simulations *OASYS*.

Due to the larger fluctuations of its photon beam properties, the photon transport system of a FEL uniquely differs from that of a synchrotron radiation source by the need for completely new photon diagnostics with the capability to continuously monitor the beam properties, ideally shot-by-shot. Novel devices to monitor intensity, beam position and pointing, but also spectrum, wavefront, beam polarization as well as pulse length and synchronization, perform these diagnostics ideally in a non-invasive way to provide real-time information to the experimentalists and to the operators for machine performance optimization. This is the subject of the PhotonDiag workshop series on ‘FEL Photon Diagnostics, Instrumentation and Beamline Design’. To face these challenges, in 2010, as part of the IRUVX EU project, a first workshop was held in Hamburg, Germany. Since 2015, the PhotonDiag series has been organized regularly, typically every two years, as an activity of FELS OF EUROPE – the collaboration of European FEL and advanced short-pulse light source (SPS) facilities.

The combination of the two workshops gave the opportunity to focus on all topics of the two workshops in an integrating way benefiting both the FEL community and the synchrotron community with their new focus on diffraction-limited storage rings (DLSRs) currently under construction or in commissioning and first user operation around the world. More than 110 participants from 13 countries, coming from FEL facilities, synchrotrons, universities, private and public institutions, and companies, gathered together to discuss and present the latest achievements in this multi-faceted field. Forty-two presentations, 40 posters and 11 industrial sponsors made the event a unique forum to assess the current state of the art and to envision the next-future developments in X-ray optics, transport, photon diagnostics and science. ▸

## Figures and Tables

**Figure 1:**
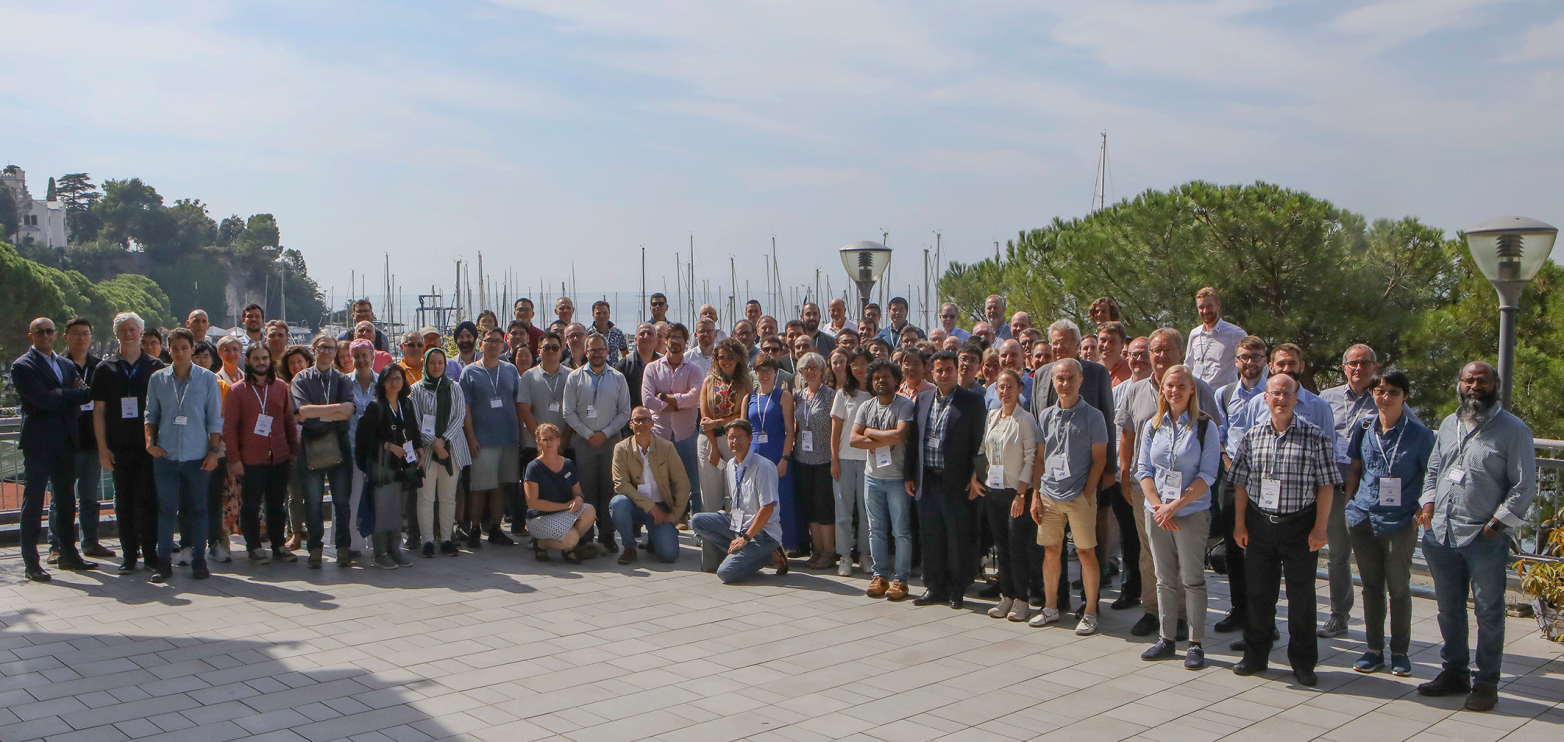
Group photograph from the PhotonMEADOW2023 workshop.

